# Single Molecular Demonstration of Modulating Charge Inversion of DNA

**DOI:** 10.1038/srep38628

**Published:** 2016-12-08

**Authors:** Yanwei Wang, Ruxia Wang, Bozhi Cao, Zilong Guo, Guangcan Yang

**Affiliations:** 1 School of Physics and Electronic Information, Wenzhou University, Wenzhou, 325035, China

## Abstract

Charge inversion of DNA is a counterintuitive phenomenon in which the effective charge of DNA switches its sign from negative to positive in the presence of multivalent counterions. The underlying microscopic mechanism is still controversial whether it is driven by a specific chemical affinity or electrostatic ion correlation. It is well known that DNA shows no charge inversion in normal aqueous solution of trivalent counterions though they can induce the conformational compaction of DNA. However, in the same trivalent counterion condition, we demonstrate for the first time the occurrence of DNA charge inversion by decreasing the dielectric constant of solution to make the electrophoretic mobility of DNA increase from a negative value to a positive value. In contrast, the charge inversion of DNA induced by quadrivalent counterions can be canceled out by increasing the dielectric constant of solution. The physical modulation of DNA effective charge in two ways unambiguously demonstrates that charge inversion of DNA is a predominantly electrostatic phenomenon driven by the existence of a strongly correlated liquid (SCL) of counterions at the DNA surface. This conclusion is also supported by the measurement of condensing and unraveling forces of DNA condensates by single molecular MT.

Charge inversion (CI) is a counterintuitive phenomenon in which a macroion in solution of multivalent counterions attracts more opposite charges in excess of its own nominal charge so that its effective charge changes its sign. DNA is a highly charged polymer, and is tightly packaged from viruses to eukaryotic cells in order to store, transport and preserve the genetic material. Thus, depending on the properties of the surrounding electrolyte, the character of interaction between two identical DNA molecules can change from repulsion to attraction^1^,^2^, which is beyond a simple neutralization effect. On the other hand, inversion of the negative charge of a DNA double helix by its complexation with a positive polyelectrolyte can be used for gene delivery since the positive charge of the complex facilities its contact with a typically negative cell membrane making penetration more likely. Therefore, understanding the attraction between like-charged macromolecules such as DNA^3^ and their charge inversion^4^,^5^ is of fundamental interest in biology and potential therapeutic applications. However, the underlying microscopic mechanism of attraction between like-charged macroions and their charge inversion is still controversial although these effects have been observed in various systems from simple colloids to intricate protein-DNA complexes by various experimental approaches. Two driving mechanisms have been proposed: pure electrostatic interaction from a strong correlation effect and specific chemical adsorption of counterions.

In recent years, some new single molecular approaches have been used to explore the issue to get more insights. For example, Lemay group^6^ observed the reversal of the polarity of charged surfaces in water upon the addition of trivalent and quadrivalent ions by atomic force microscopy. They found that the bulk concentration of multivalent ions at which CI occurs depends almost only on the valence of counterions. Then they turned to DNA system and demonstrated the CI of DNA by multivalent ions through measuring electrophoretic mobility (EM) of condensed DNA by dynamic light scattering (DLS). Measuring the multivalent-ion induced condensation of a single DNA molecule by using magnetic tweezers (MT), they further showed that CI is related to the condensation by modulating the barrier for condensate nucleation^7^. For DNA systems, only quadrivalent counterions of high concentration can lead to the CI, which is deviated from the theoretical prediction of strong correlation theory significantly. Recently, optical tweezers (OT) were employed to study the electrophoretic and the electro osmotic motion of a single colloid immersed in electrolyte solutions of different valences, and it is found that at low colloidal charge densities, ion correlation effects alone do not suffice to produce mobility reversal^8^. On the other hand, Lösche group demonstrated the CI of a lipid monolayer at ultralow electrolyte concentrations, much lower than the predicted of ion-ions correlation theories. They suggested that transverse electrostatic correlations between mobile ions and surface charges might play an important role in the process^9^. Using streaming currents, Dekker group observed that CI occurs in rectangular silica nanochannels at high concentrations of divalent ions^10^. Regardless the accumulating observations and theoretical analysis, we need more experimental study to clarify the dominant driving mechanism for CI.

CI of DNA induced by counterions is also characterized through extensive molecular dynamics simulations^11^. The simulation clarifies the difference between the inversion of the electric charge and the electrophoretic motion of DNA resulting from a complex interplay of electrostatics and hydrodynamics. The effect of dielectric constant and finite ion size also modify ion-mediated forces between DNA molecules and are quantitatively investigated by the Monte Carlo method^12^. Although both strong correlation theory and numerical simulation predict the CI of DNA upon the high concentration of trivalent or quadrivalent counterions, it has not been observed for DNA in trivalent solution regardless its high concentration.

In the present study, we demonstrate the modulating of DNA CI by adjusting the dielectric properties of solutions. We make CI happen in trivalent counterion solution by adding ethanol to decrease the dielectric constant of medium, which is realized in trivalent counterion solutions of spermidine ([C_7_N_3_H_22_]^3+^) and tripoly-lysine (K3). Meanwhile, we also show the opposite process to neutralize DNA CI by adding amino-carboxylic acids (glycine, 6-aminocaproic acid (6-A)) to the solution of counterions having 4 or more valences, such as spermine ([C_10_N_4_H_30_]^4+^), tetrapoly-lysine (K4) and octapoly-lysine (K8) so to raise its dielectric constant. It is remarkable that adjusting the dielectric constant of the medium can be achieved in two ways through adding ethanol or zwitterionic species (ZS) to the solution of DNA. The electrostatically related condensing and unraveling forces of DNA-ion complex are measured by a single molecular MT. We found that dielectric constants of the solution also modulate the condensing and unraveling forces significantly.

## Results

### Measurement setting

**Figure 1:**
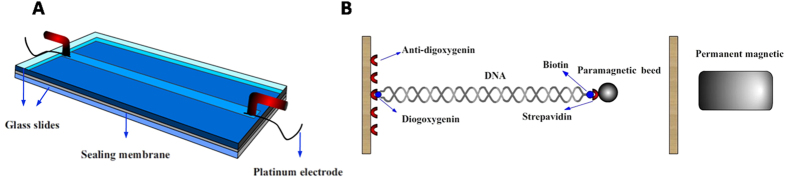
The schematic of single molecular experiments. (**A**) Single molecule electrophoresis slot, (**B**) Bead-DNA-sidewall constructs of MT.

EM of DNA in various ion conditions is measured by using DLS to observe the effect of CI. To make the results more consistent, single molecular electrophoresis (SME) measurements have been carried out by a home-made electrophoresis slot (40 mm × 3 mm × 0.1 mm), as shown in Fig. 1A, which is made by bonding two glass slides sandwiched two layers of sealing membrane. Two platinum electrodes are imbedded at the both end of slot, and a voltage is applied between the electrodes for electrophoresis in a assumed uniform electric field due to the very narrowchannel. An electron intensified charge-coupled device (ECCD) camera is used for video recording, and the EM of fluorescence dyed single DNA can be measured by analyzing the video. The single molecular force spectroscopy is performed by a home-made MT, in which aside wall-DNA-paramagnetic bead structure in the cell is shown in Fig. 1B. The distance between the bead and the surface of sidewall corresponds to the extension of DNA.A single DNA molecule can be picked out by matching the experimental force-extension curve to the known pattern of single DNA satisfying the wormlike chain model^13^. The applied force stretching DNA is calculated according to Brownian motions of the microsphere in the direction perpendicular to DNA extension. And the analysis of extension is determined by a tracking algorithm of fast Fourier transform-based correlation techniques^14^. The condensing force is defined as the force when the first contraction step in DNA extension-time curves occurred.

### Modulation of DNA electrophoresis mobility in trivalent or quadrivalent counterion solutions by dielectric properties

In a high-concentration multivalent electrolyte, the theory of counterions correlation predicts an inversion of the DNA’s electric charge^15^, which might be directly demonstrated through the motion reversal of DNA in solution being applied an external electric field. EM of DNA, μ, reflecting the net charge of DNA and the counterions condensed at its surface, can be measured by DLS in solution.

**Figure 2:**
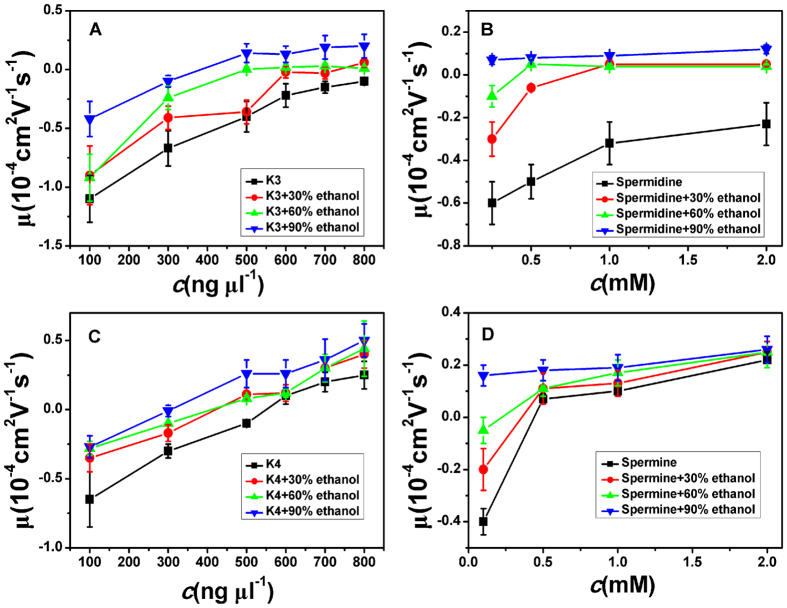
Electrophoresis mobility μ, of condensed DNA as a function of K3, K4, spermine and spermidine concentration in 1 mM TRIS and various mixture of ethanol.

The measured electrokinetic properties of DNA in trivalent and quadrivalent ion solution are shown in Fig. 2, in which the EM of condensed DNA is plotted versus the concentrations of counterions. Two species of counterion with the same valance were used for consistency: spermidine and K3 for trivalent ions while spermine andK4 for quadrivalent ions. In trivalent K3 and spermidine aqueous solution, DNA does not invert its charge even at the highest electrolyte concentration, being experimentally accessible (800 ng μl^−1^, 2 mM), as shown in Fig. 2A,B. However, if we adjust the dielectric properties of solution, the scenarios of electrokinetics of DNA might be changed drastically. Since the dielectric constant of aqueous solution is about 80, we lower the dielectric constant by adding ethanol of dielectric constant 25 to the solution. In the both cases of K3 and spermidine, the curves of EM shift entirely toward the zone of positive value. In other words, the ethanol promotes the charge neutralization of DNA. We can see that μ becomes slightly positive, implying CI, when ethanol of high concentration is added to spermidine-DNA solution. In contrast, the CI does not occur at the highest concentration of spermidine or K3 absence of ethanol. For example, when the concentration of spermidine is 2 mM, μ is about −0.23 (in units of 10^−4^ cm^2^V^−1^s^−1^, the same unit is used for mobility in the following and will not be indicated for clarity).When ethanol (30%, 60%,90%) is introduced to the DNA-spermidine solution, EM of DNA correspondingly goes up to 0.05, 0.04 and 0.12.In the case of K3, this kind of EM promotion is more evident as we can see in Fig. 2A. It is notable that this is the first observation of the CI of DNA in a trivalent counterion solution.

As for quadrivalent spermine in 1 mM TRIS, the EM of DNA becomes positive from a negative value at the concentration of 0.5 mM, implying CI of DNA at this critical concentration. And for quadrivalent K4, as shown in Fig. 2C, the switching of DNA EM occurs at 600 ng μl^−1^ of K4 concentration. When the concentrations of counterions go up further, the corresponding positive mobility in both cases increase accordingly and finally reach some saturated values. For example, as shown in Fig. 2C,D, the saturated EM is about 0.25 in K4 solution and 0.22 in spermine solution. When ethanol is added to K4 and spermine solution, the EM curves of DNA are generally promoted toward the positive region. For example, in spermine solution of 0.1 mM concentration, when adding ethanol ofvolume fraction30%, the EM goes up from −0.4 to −0.2, and it increases continually to −0.05 and 0.16when the volume fraction of ethanol is 60%and 90% respectively. The last value even becomes positive.

**Figure 3:**
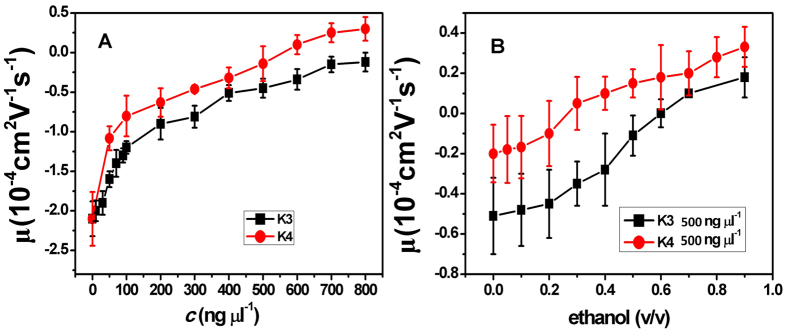
(**A**) Electrophoresis mobility μ, of condensed DNA measured by SME as a function of K3 and K4 concentration in 1 mM TRIS. (**B**) EM of condensed DNA as a function of ethanol concentration in a fixed K3 and K4 concentration of 500 ng μl^−1^.

For consistency, we measured the EM of DNA under various solution conditions by SME directly, as shown in Fig. 3, comparing with the corresponding results of DLS. In Fig. 3A, we plot the measured mobility of condensed DNA versus the concentration of K3and K4 by using SME. We can see that the mobility of DNA increases and finally reaches a saturation value without changing its sign when raising the concentration of K3, implying no CI in the case. In contrast, K4 can lead to CI of DNA. We can see that the DNA mobility continually increases with K4 concentration and finally changes its sign. In Fig. 3B, we added different volume fraction concentration of ethanol to K3-DNA and K4-DNA solution, when fixing the concentration of K3 and K4 to 500 ng μl^−1^. Apparently, EM increases with the ethanol concentration and switches its sign when volume fraction of ethanol reaches about 30% (K4) or 60% (K3). The present results are consistent with the measurement of DLS above mentioned.

**Figure 4:**
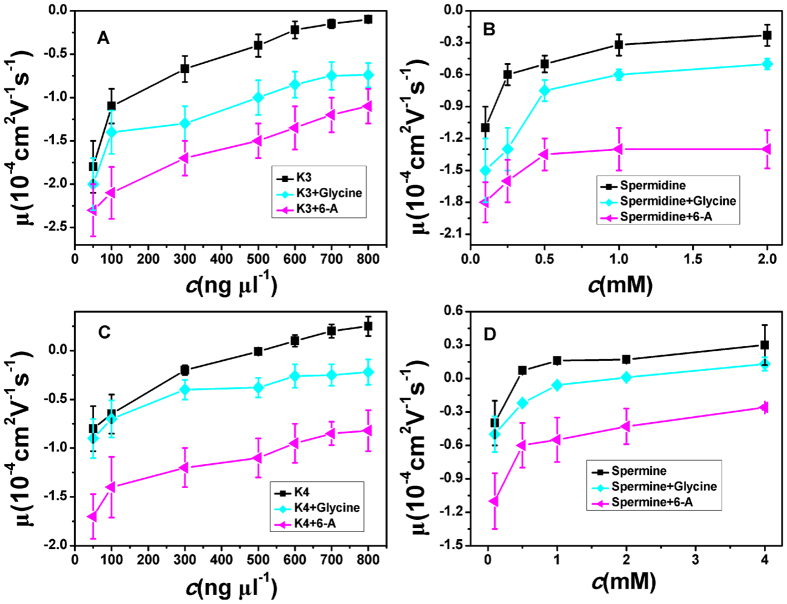
Electrophoresis mobility, μ, of condensed DNA as a function of K3, K4, spermine and spermidine concentration in 1 mM TRIS with various zwitterions.

In the above measurements, we lowered the dielectric constant of medium to help CI of DNA occurring more easily in the same ion environment. It is strongly suggested that its physical driving force is electrostatic interaction. To rule out the specific interaction between ethanol and DNA, we can execute a similar procedure but increasing the dielectric constant of solution. To do so, we add different glycine, 6-A to the aqueous solution so that the dielectric constant of medium has an increment depending on the concentration of these amino-carboxylic acids. The measured EM by DLS is shown in Fig. 4, in which glycine and 6-Aare added to variouselectrolytes. We can see that all the mobility of DNA condensates shift in the negative direction relative to the values in free ofZS. For example, as shown in Fig. 4D, when the concentration of spermine is 4 mM, DNA mobility μ is about 0.3 in absence of ZS. After ZS (glycine 1 M, 6-A 1 M) were added to DNA solution, it decreases to 0.13 and −0.26 respectively. In other words, the occurred CI is canceled out reversely since EM changes its sign from positive to negative.

### Modulation of DNA electrophoresis mobility induced by highly charged macromolecules

**Figure 5:**
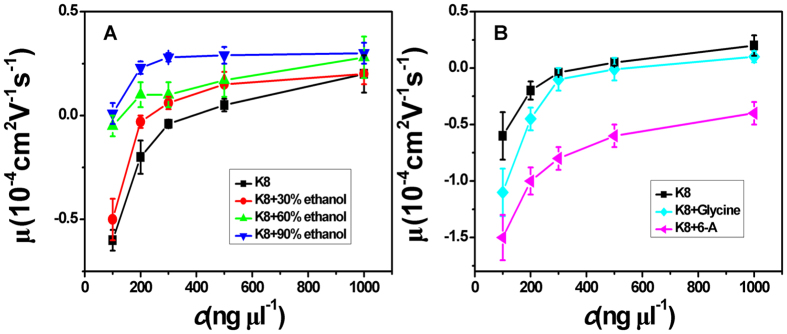
Electrophoresis mobility, μ, of condensed DNA as a function of K8 concentration in 1 mM TRIS with various ethanol (30%, 60% and 90%) and zwitterions (1 M).

To test the generality of modulation of DNA CI, we turn to the systems composed of DNA and highly charged biomacromolecules. In recent years, poly-L-lysine (PLL) mediated DNA condensation has been studied as a model for DNA compaction to nanoparticles and for the use of DNA nanoparticles for gene delivery applications^16^. K8 is double charged in solution, comparing with quadrivalent ions, was chosen to study the modulating effect on λ-DNA EM by ethanol and ZS.As shown in Fig. 5, the DNA mobility in K8 electrolytes is promoted by adding ethanol and suppressed by introducing ZS in solution. It is noteworthy that we used molar concentration in the present study, following the convention of Besteman group^7^, who have shown that CI accompanies DNA condensation by multivalent ions. Another commonly used concentration unit is charge ratio in literature. In fact, we can easily convert the molar concentration into charge ratio. For example, if we add DNA to K8 solution and both the final concentration of DNA and K8 are 1 ng μl^−1^, then the charge ratio of K8 via DNA is 2.3. If keeping the same concentration of DNA, when the concentration of K8 is 100 ng μl^−1^, then the charge ratio of K8 via DNA is 230. In our experiment, we use molar and weight concentration. From the Fig. 5, we can see that the critical concentration of K8 for DNA CI is 500 ng μl^−1^ in free of ethanol, and it decreases to 300 ng μl^−1^ in presence of 60% ethanol in solution. Once again, ZS play the role of suppressing CI of DNA. When adding 1 M 6-A to K8-DNA solution, the inverted DNA EM is switched back from positive to negative.

For consistency, we show DNA EM induced by K8 measured by SME in Table 1. The DNA mobility in K8 solution increases from negative to positive with the increment of its concentration. When we added ethanol (10%,20%,30%) to DNA-K8 mixture, with a fixed concentration of K8 of 300 ng μl^−1^, the DNA mobility varies from −0.12 to −0.09, 0 and then 0.20 respectively. In contrast, when we added Glycine (1 M) and 6-A (0.5 M, 1 M) to DNA-K8 mixture, with a fixed concentration of K8 1000 ng μl^−1^, the mobility goes down from 0.35 to 0.30, and −0.28 to −0.46 respectively. Therefore, the charge neutralization and inversion of DNA is promoted by mixing with ethanol and suppressed by mixing with ZS. The key point is that ethanol can change the sign of DNA EM from negative to positive and ZS can switch the sign of DNA EM from positive back to negative.

In our experiment, we adjust the dielectric property of solution by mixing two components having different dielectric constants. The dielectric constant of a binary solution is related to the concentrations and ratio of its constituents, and can be calculated analytically^17^. For example, when adding 10% volume fraction ethanol to water solution, the relative electric constant of solution varies from about 80 to 74.5. While 90% volume fraction ethanol is used, the dielectric constant of solution goes further down to 30.5. In the case, coulombic interaction is enhanced to 2.62 times of its value in aqueous solution. It is the enhanced electrostatic interaction promoting CI and condensation of DNA.

**Figure 6:**
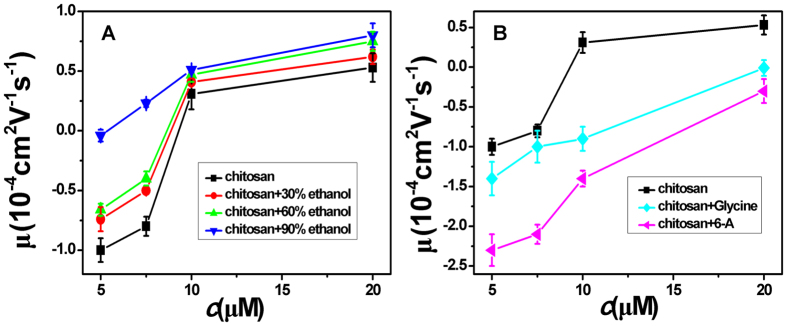
Electrophoresis mobility, μ, of DNA as a function of K8 in 1 mM Tris and 1 mM Hepes.

The behavior of buffers might also have some influence to electrostatic interaction of DNA and solvents. To clarify the buffer effect, we measured the EM of DNA under the same cation condition but using different buffers (Tris, Hepes), as shown in Fig. 6. We can see their slight difference but the same trend varying with the cation concentration. Therefore, our conclusion is still valid for different buffers.

Charge neutralization or CI usually accompany with conformal change of DNA induced by counterions. Our previous experiments show that DNA collapse induced by ethanol and confirmed the A-B transition of DNA in ethanol^18^. On the other hand, ethanol can change the dielectric constant of solution. The enhanced electrostatic interaction promotes the condensation and CI of DNA. Our focus is the modulation of CI of DNA. When CI occurs, the conformal states of DNA all are collapsed or compacted. Before and after CI, the conformational change of DNA complex is slightly. There are possible inhomogeneity of the condensed cations along the DNA axis, leading to the patterns of alternating overcharged and normal states^19^. In the present study, our main focus is the CI of the whole DNA complex while the locality is not addressed in detail. Solvent release upon the complexation of DNA and cations in the different media results in the increase of volume and entropy^20^,^21^. Because of the charge neutrality of solvents, it might have not significant influence on the CI of the whole complex.

### Condensing force of DNA complex

CI and like-charge attraction are two distinct counterintuitive phenomena of electrostatics in solution, and are closely relevant. The latter play a significant role in various types of macromolecular organization in charged systems, from clustering in colloidal suspensions to precipitation of flexible polyelectrolytes^22^. For DNA system, like-charge attraction leads to DNA compaction or condensation, in which condensing force is closely related to its charge neutralization. Since Lemay’s group investigated the folding and unfolding processes of single double strand DNA chains under various ionic conditions^7^, we focus on the role of dielectric property of solution.

The experimental procedure is similar to the measurement in ref. 39. We used a home-made MT to pull the DNA- condensing agent complexes in a flow cell, as shown in Fig. 1. After adding a condensing agent, we can see the tethered DNA compaction and measure the applied force simultaneously. Condensing force (*F*c) is defined as the force when the first contraction step in DNA extension-time curve occurred. When a magnetic bead is near to the sidewall with the compacted DNA, we can move the magnet to the cell to unravel the DNA condensate, in which the pulling force disassembling the DNA condensate is called unraveling force (*F*_U_), whose statistical mean values are presented in the paper.

**Figure 7:**
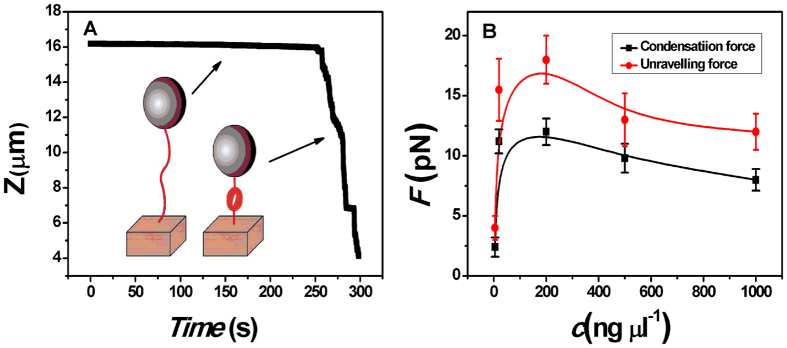
The curve of condensation, condensing and unraveling forces. (**A**) DNA extension-time curve measured by MT in DNA condensation process with K8. (**B**) *F*_*C*_ and *F*_*U*_ of DNA in K8 solution.

Figure 7A presents a typical extension-time curve of condensing of DNA. The black curve in Fig. 7B demonstrates that *F*c varies with K8 concentration. From the curve of *F*c, we can see that it initially increases rapidly and then reaches its peak around 200 ng μl^−1^ K8 concentration, which is corresponding the zone of most neutralized charge of DNA, and finally decreases accompanying with the CI of DNA. The red curve in Fig. 7B denotes *F*_U_, which shows the same trend with *F*c, while its corresponding value is bigger than *F*c. In the condensing force measurement, 20 samples are used for statistics.

**Figure 8:**
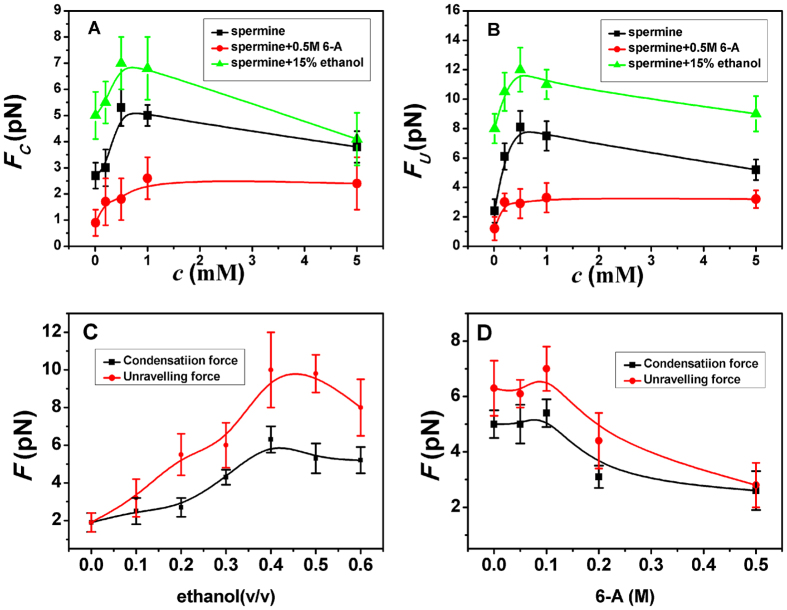
*F*_*C*_ and *F*_*U*_ of DNA in various solvent condition measured by MT. (**A**) *F*_*C*_ of DNA indifferent concentration of spermine and spermine in 15% ethanol and 0.5 M ZS. (**B**) *F*_*U*_ of DNA at the same condition as (**A**). (**C**) *F*_*C*_ and *F*_*U*_ in spermidine (1 mM) and different concentration of ethanol. (**D**) *F*_*C*_ and *F*_*U*_ in spermine (1 mM) and different concentration of 6-A.

Table 2 shows *F*c and *F*_U_ as a function of ethanol and different ZS electrolytes concentrations with a fixed K8 concentration (1000 ng μl^−1^). We can see that both *F*c and *F*_U_ increase with ethanol concentration but decrease with the concentration of 6-aminohexanoic acid. This is related to charge screening of DNA as follows: the charge of DNA is most neutralized or slightly inversed at free of ZS, as we can see in Fig. 5, which corresponds to the maximal condensing or unraveling force. After ZS is added to the solution, the charge of DNA becomes more and more negative, thus both *F*c and *F*_U_ go down gradually. In general, the fully neutralized DNA complex corresponds to a maximal condensing or unraveling force because of the least Columbic repulsion among its segments. A similar analysis can be applied to the cases of K4 (Table 3), K3 (Table 4), spermine and spermidine (Fig. 8).

In general, the DNA pulling shows that the maximal condensing or unraveling force corresponds to the completely neutralization of DNA charge, under or over compensation of charge results in descending of the force due to the enhancing Columbic repulsion.

## Discussion and Conclusion

CI cannot be explained by conventional mean-field theories of charge screening. Two possible mechanisms have been suggested: specific chemical adsorption and strong correlation effect. In the chemical adsorption mechanism, an assumed specific interaction exists between the counterions situated in the Stern layer and the surface being screened, which reduce the free energy of these ions. This binding is expected to depend on properties of the ions such as their size, chemical composition, surface structure and valence. The strong correlation effect is driven predominantly by electrostatic interactions^23^. In fact, if spatial correlations between discrete ions are accounted for, the chemical potential of the Stern layer can be significantly lowered, and a highly correlated ionic system can be formed with the loss of entropy entailed under the condition of room temperature. However, for multivalent counterions and sufficiently high surface charge densities, this is sufficiently compensated by the corresponding gain in electrostatic energy, leading to CI^24^. In that case, it is assumed that counterions form a two-dimensional SCL at charged surfaces, which in turn accompanies and influences counter-ion-induced like-charge attraction^2^. In this mechanism, DNA condensation is a purely electrostatic phenomenon driven by the existence of a SCL of counterions at the DNA surface and predicted to take place if its effective charge is close to zero^25^,^26^. The same theoretical argument predicts that multivalent counterions overcompensate the DNA charge at high counterion concentration^24^, in turn destabilizing the condensates^2^. An alternative proposed mechanism also including correlations is “electrostatic zipper” model, which assumes that the charge pattern on DNA does not comprise negative charges only but also carries a fraction of irreversibly adsorbed cations mostly in the major groove. The dressed DNA molecules condense with an exponentially decaying attraction between two DNA segments aligned side by side.

In addition to electrostatics and osmotic pressure, chemical interactions between ions and specific binding sites on DNA can also be important^27^. The effects are also attributed to differences in ion size and geometry. Ion hydration and site-specific binding also contribute. Additional considerations involve the structure of the underlying surfaces, such as the geometry of the grooves and the helical pitch of the backbone. Ion bridging may also be relevant. Finally, hydration forces, although not explicitly connected with ion distributions, must also be considered.

There might be a few other effects responsible for the phenomenon, including preferential solvation or hydration. The preferential solvation or hydration^28^,^29^ is related with the DNA condensation since the local distribution of solvent molecules around DNA complex can deviate from the bulk distribution, leading to mutual net attraction or repulsion between molecules. Rau and his collaborators measured the thermodynamic forces between precipitated DNA helices fromthe dependence of helical interracial spacing on the osmotic pressure applied by polyethylene glycol (PEG) solutions in equilibrium with the DNA phase. They found that DNA is preferentially hydrated since the alcohols examined are excluded from the condensed DNA array and strongly affect the osmotic stress force curves^30^,^31^,^32^. Therefore, the preferential solvation or hydration might simultaneously play a role on charge inversion of DNA complex.

In the framework of Poisson-Boltzmann equation, most of the charge of macromolecules can be neutralized by counterions in solution^33^,^34^, Based on the mean field theory, only 92% of negative charge of DNA can be neutralized with quadrivalent counterions in physiological conditions. Thus, CI cannot be explained by conventional mean-field theories of charge screening. Among the possible mechanisms of specific chemical adsorption and strong correlation effect, based on the experimental data here, we can draw a conclusion the CI in DNA system is driven predominantly by correlated electrostatic interactions. Although the dielectric constant seems to be the main parameter that controls the CI of DNA, one should discuss the eventual effects of the specific interaction between solvents and DNA. The local dielectric constant around the chain is thus expected to be different from that in the bulk solution. In the case of alcohols, Rau group have also shown that such exclusion mechanisms can affect the DNA precipitation by multivalent counterions such as spermidine^30^. Similarly to glycine betaine, the distribution of the zwitterions used in this study around DNA is probably not homogeneous, and exclusion from the DNA chain may be expected^35^. However, by the use of various solvent mixtures, it have been shown that the dielectric constant of the solvent is the key factor that determines the conformational behavior of single DNA molecules in solution^36^. It is reasonable to assume the conclusion is also appropriate for the electrostatic interactions of DNA in solution. However, we only consider the contribution of dielectric properties for simplicity, even if other effects, such as preferential hydration, influence the interactions between solvents and DNA. As pointed by Todd^37^, a change in dielectric constant or solute exclusion are intertwined. Our explanation is tentative and further theoretical study is needed to include all the mechanisms.

In summary, we demonstrated the occurrence of DNA CI in trivalent counterion solution by adding ethanol to lower the dielectric constant of solution while it has not been observed in normal aqueous solution. In the meanwhile, we accomplished modulation of EM of DNA in two ways by decreasing or increasing dielectric constant of solution. We unambiguously demonstrated that CI of DNA is a purely electrostatic phenomenon driven by the existence of a SCL of counterions at the DNA surface. Through observing multivalent-ion induced condensation of a single DNA molecule by single molecular MT, we further showed that condensing force of DNA was affected significantly by dielectric constant of the solution, supporting the electrostatic driving mechanism of CI of DNA.

## Methods

### Materials

Double strands λ-phage DNA (48502 bp) for MT and EM measurements was purchased from New England Biolabs company and did not go through purification when it is used, which the stoke solution was prepared in 1 × TE buffer (10 mM Tris-HCl (pH = 8.0) and 1 mM EDTA) and DNA concentration is 500 ng μl^−1^. The chemical compounds (All ions, ZS (glycine, 6-aminocaproicacid), bovine serum albumin (BSA), chitosan oligosaccharide lactate, and hydroxylmethylaminoethane (TRIS)) were purchased from Sigma-Aldrich and used as received. Ethanol was purchased from Jiani chemical technology company (Wuxi, China). YOYO-1 fluorescent dye was purchased from Haoran biological technology company (Shanghai, China). K3, K4 and K8 were ordered from Qiangyao biological technology company (Shanghai, China). Measurements were done in a 1 mM TRIS buffer at pH8.0 with varying concentration of mixing counterions. Solutions were made with 18.2 MΩ deionized water purified through the Milli-Q water purification system (Millipore Corporation, USA). All experiments were repeated at least twice to ensure consistent results while taking the standard deviation as the error bar.

DNA in SME was stained with YOYO-1 fluorescent dye at a dye-base pair ratio of 1:10 in 1 mM TRIS (pH 8.0). For the MT experiments, the λ-phage DNA were prepared by covalently attaching 12 bp chemically labeled single-stranded oligonucleotides (3′biotin-cccgccgctgga and 3′digoxygenin (dig) -tccagcggcggg) to their ends as Smith group did^38^. The DNA molecules were then mixed with 2.8 μm paramagnetic beads coated with strepavidin (M-280, Dynal Biotech) for 15 mins to form bead-DNA constructs. DNA molecules carrying a microsphere at one end and dig at the other end were ready for use.

### Electrophoretic-mobility measurements and single molecular spectroscopy

Electrophoretic-mobility (EM, μ) measurements were carried out using two methods. One was carried out by using DLS device of Malvern Zetasizer nano ZS90 equipped its patented M3-PALS technique. The laser source is a He-Ne gas laser (λ = 633 nm) and the light scattering by the avalanche photodiode mounted on the goniometer arm at to the direction of the incident radiation. The DNA molecules were diluted to a concentration of 1 ng μl^−1^ in a TRIS buffer containing different concentration of various counterions or adding ethanol, glycine or 6-aminohexanoic acid to change the dielectric constant of the solution. All measurements were carried out after 5 minutes incubation at room temperature. A 1 ml volume of DNA solution was placed in the folded capillary cells and put in the sample groove of the instrument. During the measurement, the groove temperature was kept at 25 °C.

Another EM measurement was by SME. It is based on an inverted fluorescence microscope (Nikon, TE-2000E) equipped with an oil immersion objective (Nikon, 100X, N.A. = 1.49) and a ND filter slider (Nikon, 330–385/460–490/510–550 nm). A 100 W high pressure mercury lamp served as illumination source. The intensified charge-coupled device (CCD) camera (512 × 512 pixels of Cascade II512) was used for video recording, which was used for analyzing DNA EM. NIS-Element D3.1 software was used to acquire the video and analyze the data. In the electrophoresis, the DNA was stained with YOYO-1 fluorescent dye at a dye-base-base ratio of 1:10 with TRIS buffer before using. The complex samples were incubated for 30 mins at room temperature in the dark before measurement. The EM measurements were conducted in the TRIS buffer with different concentration of the mixing counter-ions. The final concentration of DNA was about 0.4 ng μl^−1^.

The MT we used is similar to the one described by Sun *et al*.^39^. A polished sidewall of flow chamber was treated with anti-digoxygenin for the first procedure and then was rinsed with PBS containing 5 mg ml^−1^ bovine serum albumin (BSA) at pH 8.0. DNA-bead construct was then flowed into the cell to form a side wall-DNA-paramagnetic bead structure. The distance between the bead and the surface of the sidewall can be measured as the extension of DNA. The applied force was calculated according to Brownian motions of the microsphere in the direction perpendicular to the DNA extension. The analysis of the extension was determined by a tracking algorithm of fast Fourier transform-based correlation techniques^14^. Single molecule DNA condensation measurements were carried out by measured the DNA extension in time while lowing the force in discrete steps.

Before condensation force (*F*c) measurements, the BSA buffer was removed by rinsing with 10 ml TRIS. This was done because BSA influenced the condensation dynamics, presumably owing to BSA clustering and adhering to DNA. After checking a single suspending λ-DNA, we exerted the maximum force to the DNA. Then different concentrations of agents were injected to the flow cell incubating for 10 min and the elastic response of DNA as a function a time was recorded and analyzed at different forces.

## Additional Information

**How to cite this article**: Wang, Y. *et al*. Single Molecular Demonstration of Modulating Charge Inversion of DNA. *Sci. Rep.* **6**, 38628; doi: 10.1038/srep38628 (2016).

**Publisher's note:** Springer Nature remains neutral with regard to jurisdictional claims in published maps and institutional affiliations.

## Figures and Tables

**Table 1:** EM in fluorescent electrophoretic measurement, μ, of condensed DNA as a function of K8 concentration, c. and K8 in different solution condition containing different concentration ethanol and zwitterionic ions.

Solution	K8 (ng μl^−1^)	ethanol	glycine (M)	6-Aminocaproic acid (M)	EM (10^−4^ cm^2^ V^−1^ S^−1^)
A	50	0	0	0	−0.69 ± 0.40
B	100	0	0	0	−0.55 ± 0.25
C	200	0	0	0	−0.40 ± 0.11
D	500	0	0	0	0.10 ± 0.20
E	300	0	0	0	−0.12 ± 0.29
F	300	10%	0	0	−0.09 ± 0.16
G	300	20%	0	0	−0.00 ± 0.03
H	300	30%	0	0	0.20 ± 0.06
I	1000	0	0	0	0.35 ± 0.11
J	1000	0	1	0	0.30 ± 0.26
K	1000	0	0	0.5	−0.28 ± 0.08
L	1000	0	0	1	−0.46 ± 0.14

The buffer is 1 mMTRIS.

**Table 2:** The condensation and unraveling forces of DNA in K8 solution.

Solution	K8 (ng μl^−1^)	Ethanol	6-A (M)	Condensation force (pN)	Unraveling force (pN)
A	300	0	0	10.8 ± 1.1	14.5 ± 1.1
B	300	30%	0	11.9 ± 1.1	15.3. ± 1.4
C	300	50%	0	12.0. ± 1.5	16.6. ± 1.6
D	1000	0	0	8.0 ± 0.8	12 ± 0.8
E	1000	0	0.5	7.0 ± 0.5	10 ± 1.1
F	1000	0	1	5.3 ± 0.6	6.2 ± 1.0
G	1000	0	1.5	3.5 ± 0.5	5.4 ± 1.1
H	1000	0	2	1.1 ± 0.2	3.0 ± 0.8

**Table 3:** The condensation and unraveling forces of DNA in K4 solution.

Solution	K4 (ng μl^−1^)	ethanol	6-Aminocaproic acid (M)	Condensation force (pN)	Unraveling force (pN)
A	100	0	0	2.9 ± 0.5	3.1 ± 1.2
B	300	0	0	5.9 ± 1.2	6.0 ± 1.5
C	300	30%	0	6.8 ± 1.3	8.0 ± 1.7
D	300	50%	0	7.7 ± 1.1	8.9 ± 0.9
E	800	0	0	4.8 ± 0.9	5.2 ± 1.0
F	800	0	1	3.2 ± 0.2	3.5 ± 1.2
G	800	0	2	2.8 ± 1.0	3.5 ± 0.8
H	1000	0	0	4.3 ± 1.4	5.1 ± 1.6

**Table 4:** The condensation and unraveling forces of DNA in K3 solution.

Solution	K3 (ng μl^−1^)	ethanol	6-Aminocaproicacid (M)	Condensation force (pN)	Unraveling force (pN)
A	300	0	0	0.0 ± 0.0	0.0 ± 0.0
B	300	50%	0	0.8 ± 0.2	1.0 ± 0.8
C	800	0	0	1.6 ± 0.1	2.3 ± 0.6
D	800	0	1	1.0 ± 0.1	1.7 ± 0.1
E	1000	0 0	0	2.6 ± 0.5	3.0 ± 0.4
